# Supplementary material for: Molecular Epidemiology and Evolution of Coxsackievirus A14
Source: Viruses. 2023 Nov 26;15(12):2323. doi: 10.3390/v15122323 (PMC10748285; doi:10.3390/v15122323)
Supplement: Supplementary file 1 [file viruses-15-02323-s001.zip › Supplementary Data-1.pdf]

Supplementary data

Supplementary Table S1 Information on primers for amplifying all regions of 15 CVA14 strains.

Primer	Position (nt)	Primer sequence (5’-3’)	Orientation
0001S48		GGGGACAAGTTTGTACAAAAAAGCAGGCTTTAAAACAGCTCTGGGGTT	Forward
CVA14-872R	872-891	TTGGTGAAGTTCAGGGTGGA	Reverse
CVA14-636F	617-636	AGCTATTGGATTGGCCATCC	Forward
CVA14-1419R	1419-1438	GGGGGTGTGTGTGTTCATTA	Reverse
CVA14-815F	798-815	AACACCGTTCGTGATGGG	Forward
CVA14-1640R	1640-1657	CTGAAGATGCATAGGCCA	Reverse
CVA14-1489F	1470-1489	GTGTTGGATGCTGGTATACC	Forward
CVA14-2407R	2407-2426	GTGTCTCAGCTGGTACAACA	Reverse
CVA14-2135F	2119-2135	GCTTACACTCCACCAGG	Forward
CVA14-2974R	2974-2993	TGCCATTCATAGGTGTTGCG	Reverse
CVA14-2871F	2852-2871	ATAGCTTCCACCCCAGATGG	Forward
CVA14-3721R	3721-3740	ATGCCAACAACACCGTGTTG	Reverse
CVA14-3560F	3541-3560	AAACACTACCCAGTCAGCTT	Forward
CVA14-4470R	4470-4490	CAGGTTCAATACGGTGTTTGC	Reverse
CVA14-4307F	4288-4307	TCTGCTGCTTCACAAGAGGA	Forward
CVA14-4871R	4871-4890	TGAGTCCGTCACCTCAATGT	Reverse
CVA14-4589F	4570-4589	CTGTCGTAAGTTCCAACCAC	Forward

CVA14-5401R	5401-5420	AGGTCGCTAATAGCATCCGG	Reverse
**Primer**	**Position (nt)**	**Primer sequence (5’-3’)**	**Orientation**
CVA14-5070F	5050-5070	GACCACCCAAGTTTAGACCAA	Forward
CVA14-5863R	5863-5882	GACCTGCCTTAGTGGGAAAA	Reverse
CVA14-5742F	5723-5742	ATGCCATCAATGTTTGTGCC	Forward
CVA14-6689R	6689-6711	CTAGCATCATAACCTGAGTAGTC	Reverse
CVA14-6478F	6457-6478	AAAGGGAAATCTCGCCTGATAG	Forward
CVA14-7410R	7410-7429	GTGGATACAAATTTACCCCC	Reverse
CVA14-6911F	6892-6911	TTGTGTCCTTGGTGGAATGC	Forward
7500A		GGGGACCACTTTGTACAAGAAAGCTGGG$(T)_{24}$	Reverse

Supplementary Table S3 Nucleotide and amino identity (%) of 15 CVA14 strains compared with the CVA14 prototype strain (AY421769.1/G-14)

Name of strain	5'UTR	P1				P2			P3				3'UTR
		VP4	VP3	VP2	VP1	2A	2B	2C	3A	3B	3C	3D	
CHN_2013_BJ_30	85.3	86.9 (89.7)	83.8 (97.9)	83.3 (97.2)	81.9 (95.9)	81.7 (95.3)	82.4 (100)	84.2 (98.7)	82.5 (98.8)	93.9 (95.4)	85.2 (98.3)	84.6 (96.3)	93.8
CHN_2013_BJ_59	85.9	85.9 (89.7)	84.5 (97.9)	83 (97.2)	82.6 (95.9)	81.5 (95.3)	82.8 (100)	84.1 (98.7)	82.5 (98.8)	90.9 (95.4)	84.8 (98.3)	84.7 (96.1)	93.8
CHN_2009_HE_27	86.9	87.4 (89.7)	84.5 (97.9)	82 (96.8)	82.9 (96.2)	81.3 (95.3)	82.4 (100)	85.2 (98.4)	79.4 (98.8)	92.4 (100)	83 (97.8)	84.7 (97.8)	91.3
CHN_2013_HE_81	86.7	86.9 (91.1)	84.8 (97.5)	82.4 (98.4)	82.9 (96.6)	81.3 (94)	81.4 (98.9)	85.3 (97.8)	79.8 (98.8)	87.8 (100)	83.6 (96.7)	84 (96.7)	93.8
CHN_2019_HA_317	86.6	85 (91.1)	84.9 (97.9)	83.9 (97.6)	82.8 (95.9)	81.1 (94.6)	83.5 (98.9)	85 (98.4)	82.5 (97.6)	87.8 (100)	85.4 (98.3)	84.1 (96.3)	91.3
CHN_2013_HA_07	86.7	86.4 (89.7)	84.8 (97.9)	83.4 (97.6)	82.3 (95.9)	80.8 (94.6)	82.8 (100)	84.4 (98.7)	83.3 (98.8)	93.9 (95.4)	84.6 (98.3)	84 (95.8)	92.5
CHN_2013_HA_08	85.6	86.4 (91.1)	84.9 (97.9)	82.8 (98.4)	82.5 (96.9)	82 (94)	81.1 (98.9)	85.4 (98.1)	79.8 (98.8)	89.3 (100)	83.6 (96.1)	84.3 (96.9)	93.8
CHN_2013_HA_25	86.3	86.4 (89.7)	84.2 (97.9)	83.4 (97.2)	81.8 (95.9)	81.5 (95.3)	82.8 (100)	84.2 (98.7)	82.9 (98.8)	93.9 (95.4)	84.8 (98.3)	84.5 (96.1)	93.8
CHN_2013_HA_90	85.9	86.4 (89.7)	84.5 (97.9)	83.4 (97.6)	82 (95.9)	81.5 (94.6)	82.1 (100)	84.5 (98.4)	83.3 (98.8)	93.9 (95.4)	84.6 (98.3)	84.4 (96.3)	92.5
CHN_2013_LN_05	87	87.4 (91.1)	83.6 (97.5)	83 (96.8)	82.2 (95.9)	82.4 (95.3)	83.1 (100)	84.3 (98.7)	82.9 (98.8)	90.9 (95.4)	85.6 (98.3)	84.5 (96.1)	92.5

CHN_2013_TJ_52	86.1	86.4 (89.7)	84.4 (97.9)	82.9 (97.2)	82.2 (95.9)	80.6 (94.6)	82.1 (100)	84.4 (98.7)	82.1 (98.8)	93.9 (95.4)	85.4 (98.3)	84.1 (96.3)	93.8

**Name of strain**	**5'UTR**	**P1**				**P2**			**P3**				**3'UTR**
		**VP4**	**VP3**	**VP2**	**VP1**	**2A**	**2B**	**2C**	**3A**	**3B**	**3C**	**3D**	
CHN_2014_YN_41	86.5	87.9 (89.7)	84.2 (97.9)	83.2 (97.2)	81.9 (95.6)	81.1 (94.6)	83.1 (97.9)	84.3 (98.7)	82.9 (98.8)	95.4 (95.4)	84.8 (98.9)	84.1 (96.3)	91.3
CHN_2015_SN_61	89.9	89.4 (89.8)	84.5 (97.9)	84.1 (97.2)	82.2 (95.9)	84.6 (96)	85.1 (100)	83.7 (97.8)	83.3 (97.6)	84.8 (90.9)	84.1 (97.2)	83.9 (95.6)	92.5
CHN_2013_SC_30	85	86.9 (91.1)	84.4 (97.9)	83 (97.2)	82.6 (95.9)	81.1 (95.3)	82.8 (100)	83.9 (98.4)	82.9 (98.8)	92.4 (95.4)	85.7 (98.3)	84.7 (96.3)	92.5
CHN_2013_SC_36	86.8	86.9 (91.1)	84.5 (97.9)	82.6 (97.2)	82.5 (95.9)	81.5 (95.3)	82.8 (98.9)	84.1 (98.7)	82.9 (98.8)	93.9 (100)	85.4 (98.3)	84.4 (96.3)	92.5
