# Supplementary material for: Molecular Epidemiology and Evolution of Coxsackievirus A14
Source: Viruses. 2023 Nov 26;15(12):2323. doi: 10.3390/v15122323 (PMC10748285; doi:10.3390/v15122323)
Supplement: Supplementary file 1 [file viruses-15-02323-s001.zip › Supplementary Data-2.pdf]

Supplementary Table S2 Information on 22 full-length VP1 sequences retrieved from GenBank

GenBank number	Collection date	Country	Region
AF081304.1	1950	South Africa	/
KC879495.1	2005	Russia	/
GQ253379.1	2006	China	Shandong
LT719061.1	2007	Madagascar	Anjozorobe
JN203533.1	2007	India	/
JN203539.1	2009	India	/
JN639893.1	2010	China	Jiangsu
JN639894.1	2010	China	Jiangsu
LT719062.1	2011	Madagascar	Toliara, Tsimenatse
OK570249.1	2011	Madagascar	/
KX032521.1	2011	China	Henan
KP036482.1	2012	China	Jiangsu
KP036483.1	2012	China	Jiangsu
KX032522.1	2013	China	Henan
KX032523.1	2013	China	Henan
KX032524.1	2013	China	Henan
KX032525.1	2013	China	Henan
KX032526.1	2013	China	Henan
MG672482.1	2014	China	Senegal
LC013432.1	2014	China	Yunnan
LC411976.1	2014	China	Yunnan
LC411977.1	2014	China	Yunnan

Supplementary Table S4 Result of the worldwide spatial diffusion analyzed by SpreaD3

From	To	Bayes Factor	Posterior Probability
CHN	IN	1.11066535	0.20643729
CHN	MAD	1.93504447	0.31187569
CHN	RUS	2.10996510	0.33074362
CHN	SN	1.31369020	0.23529412
CHN	ZA	0.76560283	0.15205327
IN	MAD	2.26159908	0.34628191
IN	RUS	1.91509419	0.30965594
IN	SN	3.02996287	0.41509434
IN	ZA	1.13333455	0.20976693
MAD	RUS	3.27327807	0.43396226
MAD	SN	1.75999482	0.29189789
MAD	ZA	1.24170073	0.22530522
RUS	SN	1.86577531	0.30410655
RUS	ZA	1.15619560	0.21309656
SN	ZA	1.43794502	0.25194229
IN	CHN	1.99567513	0.31853496
MAD	CHN	3.31790985	0.43729190
RUS	CHN	4.80317978	0.52941176
SN	CHN	2.13119458	0.33296337
ZA	CHN	1.85600577	0.30299667
MAD	IN	1.81723679	0.29855716
RUS	IN	1.64868120	0.27857936
SN	IN	2.51501007	0.37069922
ZA	IN	6.29867532	0.59600444
RUS	MAD	2.94779264	0.40843507
SN	MAD	2.28385153	0.34850166
ZA	MAD	2.46748290	0.36625971
SN	RUS	1.88540817	0.30632630
ZA	RUS	2.58757160	0.37735849
ZA	SN	3.53337363	0.45283019

Supplementary Table S5 Result of the spatial diffusion in mainland China analyzed by SpreaD3

From	To	Bayes Factor	Posterior Probability
SD06	JS10	10.82867852	0.46836848
SD06	JS12	0.83010099	0.06326304
SD06	YN14	0.83010099	0.06326304
SD06	LN13	1.08365502	0.08102109
SD06	TJ13	0.87690749	0.06659267
SD06	SN15	0.89258395	0.06770255
SD06	SC13	1.06752115	0.07991121
SD06	BJ13	0.93983808	0.07103219
SD06	HE13	5.74529124	0.31853496
SD06	HE09	0.87690749	0.06659267
SD06	HA13	4.61628199	0.27302997
SD06	HA11	2.81711016	0.18645949
SD06	HA19	0.95566483	0.07214206
HE09	HA13	1.46579716	0.10654828
HE09	HA11	2.31881102	0.15871254
HE09	HA19	1.48290800	0.10765816
HE09	JS10	1.13229129	0.08435072
HE09	JS12	1.51725770	0.10987791
HE09	YN14	1.44872878	0.10543840
HE09	LN13	1.18128257	0.08768036
HE09	TJ13	1.51725770	0.10987791
HE09	SN15	1.41471875	0.10321865
HE09	SC13	2.07246640	0.14428413
HE09	BJ13	1.31369144	0.09655938
HE09	HE13	1.06752115	0.07991121
JS10	JS12	1.36401824	0.09988901
JS10	YN14	1.97991623	0.13873474
JS10	LN13	1.00337306	0.07547170
JS10	TJ13	1.38087668	0.10099889
JS10	SN15	1.63884266	0.11764706
JS10	SC13	1.43170269	0.10432852
JS10	BJ13	1.67397548	0.11986681
JS10	HE13	1.23063276	0.09100999
JS10	HA13	2.14737532	0.14872364
JS10	HA11	7.73486317	0.38623751
JS10	HA19	0.90829778	0.06881243
HA11	HA19	0.73747920	0.05660377
HA11	JS12	6.51085198	0.34628191
HA11	YN14	10.35587691	0.45726970
HA11	LN13	0.37970897	0.02996670
HA11	TJ13	2.63385427	0.17647059

HA11	SN15	6.80260982	0.35627081
HA11	SC13	8.80292627	0.41731410
HA11	BJ13	50.63185766	0.80466149
HA11	HE13	0.48201255	0.03773585
HA11	HA13	1.09982790	0.08213097
JS12	YN14	2.65399621	0.17758047
JS12	LN13	0.98743218	0.07436182
JS12	TJ13	3.21915522	0.20754717
JS12	SN15	4.18855992	0.25416204
JS12	SC13	1.99833072	0.13984462
JS12	BJ13	1.72700824	0.12319645
JS12	HE13	1.08365502	0.08102109
JS12	HA13	1.24716327	0.09211987
JS12	HA19	2.12857494	0.14761376
HE13	HA13	3.50679599	0.22197558
HE13	HA19	0.93983808	0.07103219
HE13	YN14	0.92404911	0.06992231
HE13	LN13	1.74477545	0.12430633
HE13	TJ13	0.61599855	0.04772475
HE13	SN15	0.90829778	0.06881243
HE13	SC13	1.03536991	0.07769145
HE13	BJ13	0.79908108	0.06104329
HA13	HA19	0.93983808	0.07103219
HA13	YN14	1.08365502	0.08102109
HA13	LN13	1.05142616	0.07880133
HA13	TJ13	0.87690749	0.06659267
HA13	SN15	0.86126826	0.06548280
HA13	SC13	0.83010099	0.06326304
HA13	BJ13	1.09982790	0.08213097
HA13	HE13	1.41471875	0.10321865
LN13	TJ13	0.97152948	0.07325194
LN13	SN15	0.79908108	0.06104329
LN13	SC13	0.69165692	0.05327414
LN13	BJ13	1.01935225	0.07658158
LN13	HE13	15.39487821	0.55604883
LN13	HA13	7.59104857	0.38179800
LN13	HA19	1.00337306	0.07547170
LN13	YN14	0.78362606	0.05993341
TJ13	SN15	1.78044533	0.12652608
TJ13	SC13	1.58647362	0.11431743
TJ13	BJ13	1.60388616	0.11542730
SC13	BJ13	1.69160842	0.12097669
TJ13	HE13	1.34720132	0.09877913
SC13	HE13	1.81629696	0.12874584

TJ13	HA13	1.39777680	0.10210877
SC13	HA13	1.31369144	0.09655938
TJ13	HA19	2.43538121	0.16537181
SC13	HA19	2.01679280	0.14095450
TJ13	YN14	1.81629696	0.12874584
SC13	YN14	1.55177914	0.11209767
TJ13	LN13	1.43170269	0.10432852
SC13	LN13	1.28034583	0.09433962
SC13	TJ13	2.43538121	0.16537181
SC13	SN15	1.78044533	0.12652608
BJ13	HE13	0.90829778	0.06881243
BJ13	HA13	1.08365502	0.08102109
BJ13	HA19	1.36401824	0.09988901
BJ13	YN14	2.22306966	0.15316315
BJ13	LN13	0.92404911	0.06992231
BJ13	TJ13	2.14737532	0.14872364
BJ13	SN15	2.45499066	0.16648169
BJ13	SC13	4.33702730	0.26082131
YN14	SN15	3.57469620	0.22530522
YN14	HA19	14.39417225	0.53940067
SN15	HA19	2.20407177	0.15205327
